# Genetic dominance of transforming growth factor-β1 polymorphisms in chronic liver disease

**DOI:** 10.3389/fimmu.2022.1058532

**Published:** 2022-11-16

**Authors:** Xuanyan Cai, Huiyan Zha, Zhaoxu Yang, Yiwen Du, Xiaoyang Dai, Bo Yang, Jiajia Wang, Qiaojun He, Qinjie Weng

**Affiliations:** ^1^ Center for Drug Safety Evaluation and Research, College of Pharmaceutical Sciences, Zhejiang University, Hangzhou, China; ^2^ Zhejiang Province Key Laboratory of Anti-Cancer Drug Research, College of Pharmaceutical Sciences, Zhejiang University, Hangzhou, China; ^3^ The Second Affiliated Hospital, Zhejiang University School of Medicine, Hangzhou, China

**Keywords:** transforming growth factor-β1, polymorphisms, susceptibility, chronic liver disease, cirrhosis

## Abstract

Chronic liver disease (CLD) is an extremely common clinical condition accompanied by sustained inflammatory response leading to tissue damage. Transforming growth factor-β1 (TGF-β1) is known as a master immune regulator in CLDs, but the association between TGF-β1 polymorphisms and CLD risk is controversial and inconclusive, and the genetic dominance of CLDs remains unknown. In this study, the relationship between *TGF-β1* polymorphisms and CLD susceptibility is systematically analyzed based on 35 eligible studies. Individuals with the *TGF-β1*-509 allele (TT or CT) or codon 10 allele (Pro/Pro) show an increased risk of CLDs. Subgroup analyses indicate *TGF-β1*-509C/T has a significant correlation with cirrhosis and chronic hepatitis C, codon 10 is associated with chronic hepatitis B occurrence, and codon 25 exhibits a relationship with autoimmune hepatitis risk. Missense mutations in G29E, A105S, D191N, and F321L of *TGF-β1* are the genetic factors of HCC susceptibility. Furthermore, the *TGF-β1* gene expression is significantly elevated in CLD patients, and the *TGF-β1* codon 263 is located close to the region where the TGF-β1 dimerization interacts, indicating the *TGF-β1* codon 263 variant may affect the secretion of TGF-β1 by altering its dimerization. Together, our findings provide new insights into the immune regulator gene *TGF-β1* polymorphisms as susceptibility factors for CLD occurrence and regulators for TGF-β1 expression, which have implications for the regulation of immune factors during CLD development.

## Introduction

Chronic liver disease (CLD) is a continuous and gradual process caused by multifactorial etiologies including genetic factors. The pathogenesis of CLD is complicated and is related to sustained inflammatory response leading to tissue damage (1, 2). Chronic hepatotoxic injuries, such as hepatitis B virus (HBV) or hepatitis C virus (HCV) infections, autoimmune hepatitis (AIH), non-alcoholic steatohepatitis (NASH) and alcohol abuse, can severely impair liver functions, progressing to cirrhosis, hepatocellular carcinoma (HCC) or even liver failure (1). CLDs represent big challenges to the public health system worldwide, which account for more than 800 million individuals and approximately 2 million deaths per year in the world (1–3). Interestingly, the risk of CLDs varies from one individual to another (4–6), indicating that the host genetic factors might determine the susceptibility of CLDs.

Transforming growth factor-β1 (TGF-β1), a member of the TGF-β super-family, is regarded as a crucial immune regulator in a variety of CLDs (7). In the hepatocarcinogenic process, TGF-β1 functions as an anti-inflammatory cytokine that promotes immune surveillance escape in HCC, leading to cell migration and invasion (8–10). TGF-β also activates SMAD2/3 and cooperates with IL-21 activity to promote naïve CD4^+^ T cell generation of Th17 cells, contributing to NASH-associated liver inflammation and HCC (11, 12). In the pathogenesis of chronic hepatitis, TGF-β1 leads to prolonged HBV/HCV infections *via* increasing T regulatory lymphocyte activation and recruiting these cells to the infected livers (13–15). During fibrogenesis, TGF-β1 could be secreted by different immune cells, such as hepatic macrophages and regulatory T lymphocytes, and induce hepatic stellate cells to transform into proliferative fibrogenic myofibroblasts, synthesizing and secreting excess collagen (16–18).

Several studies have found that single-nucleotide polymorphisms (SNPs) of *TGF-β1* may play important roles in CLDs. Up to now, *TGF-β1*-509C/T (rs1800469) and *TGF-β1* codon 10 (rs1800470) polymorphisms have been reported to have a potential relationship with the susceptibility of HBV/HCV-induced cirrhosis or HCC (19–25). However, the findings are contradictory and inconclusive. Specifically, one study pointed out that patients with the *TGF-β1*-509 TT genotype were significantly associated with cirrhosis risk (24), while another study concluded that the *TGF-β1*-509C/T polymorphism had a limited role in predicting cirrhosis occurrence (21, 22). Similar controversial results were also found in HCC risk prediction (19, 20, 23, 25). In addition, although *TGF-β1* genetic variation occurs in cirrhosis and HCC, two etiologies of CLDs, whether *TGF-β1* polymorphisms are associated with other types of CLD risk and which polymorphisms are high genetic dominance of CLDs remain unidentified.

A series of studies found that TGF-β1 expression was significantly higher in patients with cirrhosis (26, 27), and upregulated in tumor tissues of HCC (28–30). Clinic studies showed that individuals with *TGF-β1*-509C/T or codon 10 displayed increased plasma concentration of TGF-β1, implying that *TGF-β1* polymorphisms may regulate TGF-β1 expression in CLD patients (21, 31–33). However, how these genetic variants intervene the transcription, translation, or protein 3D structure of TGF-β1 is complicated and mysterious. The investigation between *TGF-β1* genetic variants and TGF-β1 expression will have great implications for the development of CLDs.

Accordingly, we performed a systematic meta-analysis to determine the associations between SNPs of immune regulator gene *TGF-β1* and CLD susceptibility. We assessed the *TGF-β1* polymorphisms in diverse etiologies and identified a strong association of specific *TGF-β1* gene variants with different CLD types. Furthermore, we applied bioinformatic analysis to establish *TGF-β1* expression in CLDs with different etiologies and explore the potential way by which *TGF-β1* polymorphisms affect its expression. Consequently, our findings reveal a previously unidentified role of *TGF-β1* polymorphisms in predicting CLD susceptibility and the underlying mechanisms involved.

## Materials and methods

### Study selection and identification

The analysis was conducted following the Preferred Reporting Items for Systematic Reviews and Network Meta-Analyses (PRISMA) guidelines (34). We searched English-language publications in Embase, Pubmed, Web of Science, Cochrane Library, and HuGe Navigator from database inception through June 2022 that reported the *TGF-β1* polymorphisms and CLDs in humans. We used various combinations of the search terms to screen for relevant studies: ((TGFB1 OR TGF beta 1 OR Transforming Growth Factor OR TGF-β) AND (single nucleotide polymorphism OR SNP OR genetic variation OR genetic polymorphism OR Polymorphism OR Variant OR Variation OR Mutation OR Genome-wide Association Study OR Genetic Association Study)) AND (Non-Alcoholic Fatty Liver Disease OR Non-Alcoholic Fatty Liver OR Non-Alcoholic Steatohepatitis OR NASH OR NAFLD). Three investigators independently selected the references and reviewed the study abstracts and full text.

The inclusion criteria were as follows: (1) studies evaluating the association between *TGF-β1* polymorphisms and CLD risk, (2) studies including case and control populations, (3) the incidence of *TGF-β1* polymorphisms in case and control groups, and (4) studies with sufficient available data to estimate an odds ratio (OR) with its 95% confidence interval (95% CI). OR is a precise estimate of the occurrence of a specific event. The involved studies should meet all of the above criteria. Exclude any studies with errors or inconsistent data. All potentially eligible trials were considered regardless of outcomes.

### Data extraction and quality assessment

The following information was extracted for each eligible study: the name of the first author, the publication year, the country of origin, ethnicity, genotyping method, sex, disease type, sample sizes of cases and controls, and genotype number in cases and controls. In our study, we used the Newcastle-Ottawa Scale (NOS) to assess the quality (35). The NOS contains the following 3 dimensions: selection, comparability, and exposure, covered by 8 items. The overall score was nine, with higher scores indicating better quality. Studies with a score of 4-6 were deemed intermediate quality, and those with a score > 7 were deemed high quality.

### Gene expression, mutation, and transcription factor analysis of TGF-β1

The *TGF-β1* gene expression profiles were downloaded from the Gene Expression Omnibus (GEO) database: GSE202853 (HCC), GSE25097 (Cirrhosis), GSE38941 (CHB), GSE154055 (CHC), GSE159676 (AIH), GSE28619 (Alcohol hepatitis) and GSE48452 (NASH). Expression profiles of these datasets were reanalyzed using R and correlated packages (http://www.r-project.org/). We used cBioPortal (https://www.cbioportal.org/) to investigate the gene mutation of *TGF-β1* in HCC (36). The transcription factors of *TGF-β1* were downloaded from Genecard, Ominer, Promo, and TRRUST databases.

### Single-cell RNA sequencing analysis

We reanalyzed the published scRNA-seq data of human liver cirrhosis from GSE136103 (37). The raw reads were aligned to the mm10 (Ensembl 84) reference genomes, utilizing an in-house pipeline of the Cell Ranger v5.0.1 Single-Cell Software Suite from 10X Genomics. Additional analysis was then executed by the “Seurat” (v4.0.0) package for R (v4.0.4) (https://www.r-project.org/). Genes expressed in fewer than three cells in a sample were excluded, as were cells that expressed fewer than 200 genes or mitochondrial gene content > 10% of the total UMI count. In addition, cells were used for further analysis if they passed an expressed gene threshold of 4000 genes. Cluster identification was based on the 50 most significant principal components. Cell type recognition was automatically annotated by the software “SingleR” (v3.15) (38). All uniform manifold approximation and projection (UMAP) visualizations and violin plots were produced using Seurat functions in conjunction with the ggplot2.

### Structure modeling of TGF-β1

The crystal structure of residues between 1 and 29 was not solved by experiments, and we modeled it using the GalaxyHomomer method of GalaxyWeb (39, 40). The best model predicted by GalaxyWeb was applied for the following analysis.

### Statistical analysis

The Hardy-Weinberg Equilibrium (HWE) is a useful indicator of genotype frequencies within a population and whether they are based on a valid definition of alleles and a randomly mating sample, which was measured in the controls by Pearson’s chi-square test (*P* < 0.05 was considered a departure from HWE). Statistical analysis was performed by RevMan 5.3 (Cochrane Community, 2014), which generated a forest plot, pooled ORs, and 95% CIs for each risk factor. We calculated the pooled ORs and 95% CIs to assess the association between *TGF-β1* polymorphisms and the susceptibility to CLDs. The significance of the ORs was determined with the Z-test, which is used to infer the probability of difference with the theory of standard normal distribution, and *P* < 0.05 was considered statistically significant. The heterogeneity assumption was evaluated by the Cochrane *I* ^2^ test and Q-statistic (*P_het_*) (41). When the *P_het_* > 0.1 and *I* ^2^ < 50%, the fixed-effects (FE) model was adopted to calculate the pooled ORs; otherwise, the random-effects (RE) model was applied. The publication bias was assessed using funnel plots generated by RevMan 5.3. The Begg’s test (42) and Egger’s test (43) were also used to evaluate publication bias performed by Stata 15 (StataCorp 2017, Dallas, Texas, USA).

## Results

### Study selection and study characteristics

**Figure 1:**
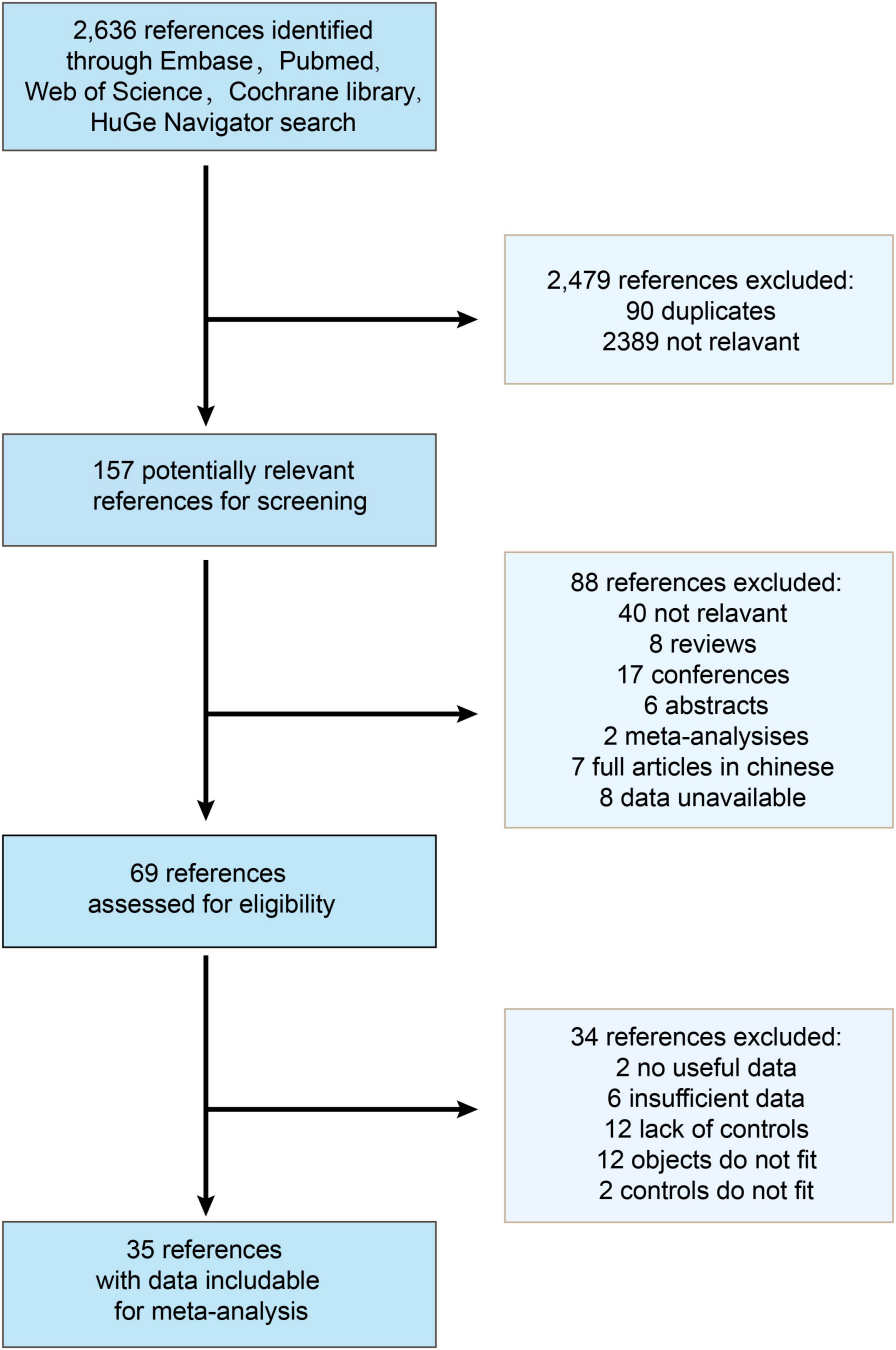
Flow chart of the study selection process. 35 studies were determined to be eligible for inclusion in the meta-analysis.

A total of 2,636 references were identified through Embase, Pubmed, Web of Science, Cochrane Library, and HuGe Navigator, of which 2,479 were excluded for irrelevance and duplication. After reviewing 157 articles, 88 articles did not satisfy the selection criteria, leaving 69 studies for detailed full-text evaluation. Ultimately, 35 studies were judged to meet the inclusion criteria for this meta-analysis (**Figure 1**).

The eligible 35 studies included 5,225 patients with a variety of CLDs, such as HCC, cirrhosis, chronic hepatitis B (CHB), chronic hepatitis C (CHC), AIH, alcoholic liver disease (ALD), and primary biliary cirrhosis (PBC). These studies were performed in countries around the globe, including China, America, India, Brazil, Spain, Italy, Iran, Mexico, Japan, Pakistan, Turkey, and Egypt, and were mixed-sex, showing the data are comprehensive and extensive. Moreover, we assessed the quality of 35 studies based on the NOS criteria (35). All of these studies exhibited comparatively high quality with a score of more than six (**Table 1**).

**Table 1:** The characteristics of included studies.

Reference	Country	Ethnicity	Genotyping method	Sex	Disease	Number		Study quality (NOS)
						Case/n	Control/n	
Suzuki, S. 2003	Japan	Asian	PCR-RFLP	Mixed	CHC	206	101	8
Kikuchi, K. 2007	Japan	Asian	PCR-RFLP	Mixed	PBC	65	71	7
Basturk, B. 2008	Turkey	Caucasian	PCR-SSP	Mixed	CHB	27	60	8
Romani, S. 2011	Iran	Caucasian	PCR-RFLP	Mixed	CHC	164	169	7
Talaat, R. M. 2013	Egypt	Egyptian	SSP-PCR	Mixed	CHB	65	27	9
Hosseini Razavi, A. 2014	Iran	Caucasian	PCR-RFLP	Mixed	CHB	220	220	8
Fabríciosilva-Silva. 2015	Brazil	Mixed-race	TaqMan	Mixed	CHC	245	189	8
Dondeti, M. F. 2017	Egypt	Egyptian	ARMS-PCR	Mixed	CHB	115	119	9
Obada, M. 2017	Egypt	Egyptian	PCR-RFLP	Mixed	CHC	150	100	9
Ghani, M. U. 2019	Pakistan	Asian	PCR-RFLP	Mixed	CHC	96	98	9
					HCC	94	98	
Sánchez-Parada. 2013	Mexico	Mixed-race	TaqMan	Mixed	CHC	38	50	8
Oliver, J. et al., 2005	Spain	Caucasian	PCR-RFLP	Mixed	ALD	165	185	8
Armendáriz-Borunda, J. 2008	Mexico	Mixed-race	PCR-RFLP	Mixed	CHC	13	30	8
					ALD	7	30	
Qi, P. 2009	China	Asian	PCR-RFLP	Mixed	CHB	196	299	8
					HCC	379	299	
Radwan. 2012	Egypt	Egyptian	PCR-RFLP	Mixed	Cirrhosis	152	160	9
					HCC	128	160	
Roy, N. 2012	India	Asian	PCR-RFLP	Male	ALD	169	108	8
Conde, S. R. 2013	Brazil	Mixed-race	PCR-RFLP	Mixed	CHB	53	97	8
Mohy, A. 2014	Egypt	Egyptian	PCR-RFLP	Mixed	Cirrhosis	40	40	9
Saxena. 2014	India	Asian	PCR-RFLP	Mixed	CHB	61	153	8
					Cirrhosis	60	153	
					HCC	59	153	
Bader El Din, N. G. 2017	Egypt	Egyptian	PCR-RFLP	Mixed	CHC	72	50	9
Brito, W. 2020	Brazil	Mixed-race	TaqMan	Mixed	CHC	97	300	7
Nomair. 2021	Egypt	Egyptian	TaqMan	Mixed	Cirrhosis	36	20	8
					HCC	34	20	
Vidigal, P. G. 2002	America	Caucasian	PCR-RFLP	Male	CHC	80	37	9
Zein. 2004	Mixed	Egyptian	PCR-RFLP	Mixed	CHC	24	45	7
		Caucasian	PCR-RFLP	Mixed	CHC	31	36	
Falleti, E. 2008	Italy	Caucasian	PCR-SSP	Mixed	Cirrhosis	188	140	9
Pereira, F. A. 2008	Brazil	Mixed-race	PCR-RFLP	Mixed	CHC	128	94	9
Wang, H. 2008	China	Asian	ARMS-PCR	Mixed	Cirrhosis	118	104	9
Paladino, N. 2010	America		SSOP	Mixed	AIH	178	189	6
Lee, J. J. 2011	Korea	Asian	SSCP	Mixed	Cirrhosis	182	119	7
Shi, H. Z. 2012	China	Asian	PCR-RFLP	Mixed	HCC	72	117	6
Xin, Z. H. 2012	China	Asian	TaqMan	Mixed	HCC	347	881	8
Maria. 2013	Mexico	Mixed-race	TaqMan	Mixed	CHC	38	50	9
Ma, J. 2015	China	Asian	PCR-RFLP	Mixed	CHC	234	375	9
					HCC	159	375	
Eskandari, E. 2017	Iran	Caucasian	ARMS-PCR	Mixed	CHB	196	198	8
Yousefi, A. 2019	Iran	Caucasian	PCR-SSP	Mixed	AIH	44	138	8

PCR, polymerase chain reaction; RFLP, restriction fragment length polymorphism; SSP, sequence-specific primer; ARMS, amplification refractory mutation system; SSOP, sequence-specific oligonucleotide probing; SSCP, single stranded conformational polymorphism; HCC, hepatocellular carcinoma; PBC, primary biliary cirrhosis; CHB, chronic hepatitis B; CHC, chronic hepatitis C; ALD, alcoholic liver disease; AIH, autoimmune hepatitis; NOS, Newcastle-Ottawa Scale.

### Genotype distributions of *TGF-β1* polymorphisms

Five *TGF-β1* polymorphisms participated in these 35 studies, including *TGF-β1*-509C/T (rs1800469), *TGF-β1* codon 10 (rs1800470), *TGF-β1* codon 25 (rs1800471), *TGF-β1*-800G/A (rs1800468) and *TGF-β1* codon 263 (rs1800472) polymorphisms. The genotypes and allele frequencies of every *TGF-β1* polymorphism distribution in the involved studies were summarized: *TGF-β1*-509C/T (**Table 2**), *TGF-β1* codon 10 (**Table 3**), *TGF-β1* codon 25 (**Table 4**), *TGF-β1*-800G/A (**Table S1**) and *TGF-β1* codon 263 (**Table S2**). According to the HWE examination, genotype distributions in controls of the overwhelming majority of studies were in agreement with HWE except for several studies. Thus, studies with HWE less than 0.05 were excluded for further meta-analysis.

**Table 2:** The genotype and allele frequencies of *TGF-β1*-509C/T distribution of included studies.

Reference	Disease	Number		Case			Control			*P* for HWE	
		Case/n	Control/n	CC	CT	TT	CC	CT	TT	Case	Control
Bader El Din, N. G. 2017	CHC	72	50	22	32	18	23	23	4	0.358	0.595
Brito, W. 2020	CHC	97	300	17	49	31	90	154	56	0.754	0.488
Conde, S. R. 2013	CHB	53	97	17	19	17	24	39	34	0.039	0.065
Dondeti, M. F. 2017	CHB	115	119	11	85	19	35	75	9	0.000	0.000
Eskandari, E. 2017	CHB	178	154	78	78	22	71	54	29	0.715	0.003
Falleti, E. 2008	Cirrhosis	188	140	50	85	53	57	61	22	0.190	0.404
Ghani, M. U. 2019	CHC	96	98	22	47	27	38	42	18	0.859	0.296
	HCC	94	98	18	47	29	38	42	18	0.893	0.296
Hosseini Razavi, A. 2014	CHB	220	220	50	116	54	65	97	58	0.416	0.082
Kikuchi, K. 2007	PBC	65	71	21	32	12	27	31	13	0.975	0.441
Ma, J. 2015	CHC	234	375	91	101	42	143	161	71	0.137	0.036
	HCC	159	375	50	67	42	143	161	71	0.051	0.036
Mohy, A. 2014	Cirrhosis	40	40	9	21	10	33	4	3	0.749	0.001
Oliver, J. 2005	ALD	165	185	64	78	23	79	85	21	0.921	0.795
Qi, P. 2009	CHB	196	299	31	101	64	50	156	93	0.396	0.257
	HCC	379	299	89	198	92	50	156	93	0.382	0.257
Radwan. 2012	Cirrhosis	152	160	34	74	44	62	68	30	0.785	0.147
	HCC	128	160	24	64	40	62	68	30	0.857	0.147
Roy, N. 2012	ALD	169	108	80	75	14	39	48	21	0.539	0.373
Shi, H. Z. 2012	HCC	72	117	24	40	8	55	53	9	0.152	0.438
Wang, H. 2008	Cirrhosis	118	104	31	53	34	29	50	25	0.272	0.706
Xin, Z. H. 2012	HCC	347	881	82	177	88	212	432	237	0.703	0.583
Saxena. 2014	CHB	61	153	8	37	16	44	94	15	0.067	0.001
	Cirrhosis	60	153	8	48	4	44	94	15	0.000	0.001
	HCC	59	153	9	39	11	44	94	15	0.013	0.001

HCC, hepatocellular carcinoma; PBC, primary biliary cirrhosis; CHB, chronic hepatitis B; CHC, chronic hepatitis C; ALD, alcoholic liver disease; HWE, Hardy–Weinberg Equilibrium; C, Cytosine; T, Thymine.

**Table 3:** The genotype and allele frequencies of *TGF-β1* codon 10 distribution of included studies.

Reference	Disease	Number		Case			Control			*P* for HWE	
		Case/n	Control/n	Leu/Leu	Leu/Pro	Pro/Pro	Leu/Leu	Leu/Pro	Pro/Pro	Case	Control
Basturk, B. 2008	CHB	27	60	2	20	5	16	31	13	0.009	0.781
Dondeti, M. F. 2017	CHB	115	119	44	70	1	40	70	9	0.000	0.004
Eskandari, E. 2017	CHB	196	198	23	118	55	46	103	49	0.001	0.567
Fabríciosilva-Silva. 2015	CHC	245	189	70	117	58	54	103	32	0.505	0.149
Falleti, E. 2008	Cirrhosis	188	140	51	95	42	49	62	29	0.859	0.257
Lee, J. J. 2011	Cirrhosis	182	119	61	79	42	35	53	31	0.099	0.238
Obada, M. 2017	CHC	150	100	42	78	30	32	47	21	0.567	0.628
Oliver, J. 2005	ALD	165	185	72	68	25	75	77	33	0.186	0.096
Paladino, N. 2010	AIH	178	189	46	65	67	55	95	39	0.001	0.863
Pereira, F. A. 2008	CHC	128	94	26	65	37	24	49	21	0.793	0.672
Romani, S. 2011	CHC	164	169	50	81	33	49	85	35	0.985	0.867
Suzuki, S. 2003	CHC	206	101	56	84	66	28	52	21	0.009	0.727
Talaat, R. M. 2013	CHB	65	27	10	44	11	0	15	12	0.004	0.046
Vidigal, P. G. 2002	CHC	80	37	29	38	13	12	21	4	0.926	0.246
Wang, H. 2008	Cirrhosis	118	104	34	53	31	25	50	29	0.272	0.706
Yousefi, A. 2019	AIH	44	138	6	7	31	27	91	20	0.000	0.000
Zein. 2004	CHC(Caucasian)	31	36	10	4	17	12	3	21	0.000	0.000
	CHC (Egyptian)	24	45	6	3	15	12	11	22	0.001	0.001

CHB, chronic hepatitis B; CHC, chronic hepatitis C; ALD, alcoholic liver disease; AIH, autoimmune hepatitis; HWE, Hardy–Weinberg Equilibrium; Leu, Leucine; Pro, Proline.

**Table 4:** The genotype and allele frequencies of *TGF-β1* codon 25 distribution of included studies.

Reference	Disease	Number		Case			Control			*P* for HWE	
		Case/n	Control/n	Arg/Arg	Arg/Pro	Pro/Pro	Arg/Arg	Arg/Pro	Pro/Pro	Case	Control	
Armendáriz. 2008 (ALD)	ALD	7	30	7	0	0	11	13	6	/	0.552	
	CHC	13	30	13	0	0	11	13	6	/	0.552	
Basturk, B. 2008	CHB	27	60	23	4	0	51	6	3	0.678	0.001	
Dondeti, M. F. 2017	CHB	115	119	96	19	0	104	13	2	0.334	0.054	
Fabríciosilva-Silva. 2015	CHC	245	189	213	30	2	161	26	2	0.417	0.420	
Falleti, E. 2008	Cirrhosis	187	140	156	31	0	127	13	0	0.216	0.565	
Hosseini Razavi, A. 2014	CHB	220	220	193	23	4	197	21	2	0.003	0.105	
Maria. 2013	CHC	38	50	34	4	0	46	4	0	0.732	0.768	
Nomair. 2021	Cirrhosis	36	20	2	32	2	4	14	2	0.000	0.064	
	HCC	34	20	10	24	0	4	14	2	0.001	0.064	
Obada, M. 2017	CHC	150	100	127	22	1	84	15	1	0.965	0.721	
Oliver, J. 2005	ALD	165	185	135	28	2	148	34	3	0.690	0.523	
Paladino, N. 2010	AIH	178	189	154	15	9	156	32	1	0.000	0.638	
Pereira, F. A. 2008	CHC	128	94	113	14	1	64	29	1	0.451	0.244	
Romani, S. 2011	CHC	164	169	145	18	1	151	16	2	0.595	0.052	
Sánchez-Parada. 2013	CHC	38	50	34	4	0	46	4	0	0.732	0.768	
Vidigal, P. G. 2002	CHC	80	37	68	11	1	34	3	0	0.480	0.797	
Yousefi, A. 2019	AIH	43	138	26	7	10	119	17	2	0.000	0.146	
Zein. 2004	CHC(Caucasian)	31	36	25	6	0	33	3	0	0.551	0.794	
	CHC (Egyptian)	24	45	21	3	0	41	4	0	0.744	0.755	

HCC, hepatocellular carcinoma; CHB, chronic hepatitis B; CHC, chronic hepatitis C; ALD, alcoholic liver disease; AIH, autoimmune hepatitis; HWE, Hardy–Weinberg Equilibrium; Arg, Arginine; Pro, Proline.

### Relationship between *TGF-β1* polymorphisms and CLD Risk

The results of CLD risk associated with three SNPs (*TGF-β1*-509C/T, codon 10, and codon 25) under five genetic models were summarized in **Table 5**. Given that less than three references were available on *TGF-β1*-800G/A and codon 263 polymorphisms, a further meta-analysis was not performed for these two variants.

**Table 5:** Main results of the meta-analysis of *TGF-β1* polymorphisms in CLDs.

SNPs	Gene model	Number of study	OR (95% CI)	*P*	Test for heterogeneity		Analysis model	Publication bias	
					*I* ^2^, %	*P_het_*		Begg	Egger
-509C/T	T vs C	17	1.25 (1.06, 1.48)	0.009	78	<0.00001	RE	0.2661	0.1428
	TT vs CC	17	1.51 (1.08, 2.11)	0.02	77	<0.00001	RE	0.387	0.1492
	CT vs CC	17	1.31 (1.09, 1.58)	0.005	49	0.01	RE	0.3031	0.2309
	CT+TT vs CC	17	1.38 (1.10, 1.73)	0.005	69	<0.00001	RE	0.2016	0.1632
	TT vs CC+CT	17	1.25 (1.00, 1.57)	0.05	65	0.0001	RE	0.5923	0.1469
Codon 10	Pro vs Leu	13	1.12 (1.02, 1.23)	0.01	18	0.27	FE	1.3307	0.7615
	Pro/Pro vs Leu/Leu	13	1.28 (1.06, 1.54)	0.01	23	0.21	FE	0.9514	0.6462
	Leu/Pro vs Leu/Leu	13	1.05 (0.90, 1.23)	0.54	35	0.1	FE	0.5022	0.1072
	Pro/Pro+Leu/Pro vs Leu/Leu	13	1.11 (0.96, 1.29)	0.15	28	0.17	FE	0.2997	0.1229
	Pro/Pro vs Leu/Leu+Leu/Pro	13	1.23 (1.05, 1.44)	0.009	29	0.15	FE	1.5723	0.3629
Codon 25	Pro vs Arg	19	1.09 (0.78, 1.52)	0.60	69	<0.00001	RE	0.7526	0.1072
	Pro/Pro vs Arg/Arg	14	0.97 (0.38, 2.51)	0.95	55	0.007	RE	1.984	0.0008
	Arg/Pro vs Arg/Arg	19	1.01 (0.73, 1.39)	0.98	55	0.002	RE	0.6488	0.5689
	Pro/Pro+Arg/Pro vs Arg/Arg	19	1.07 (0.76, 1.52)	0.68	64	<0.0001	RE	0.5515	0.2743
	Pro/Pro vs Arg/Arg+Arg/Pro	14	1.00 (0.42, 2.41)	1.00	49	0.02	RE	1.9625	0.0006

SNPs, single-nucleotide polymorphisms; OR, pooled odds ratios; 95% CI, 95% confidence interval; P, P value for Z test. I^2^, Cochrane I^2^ test; P_het_, P value for heterogeneity; Begg, Begg’s tests; Egger, Egger’s tests; RE, random-effects model; FE, fixed-effects model; C, Cytosine; T, Thymine; Leu, Leucine; Arg, Arginine; Pro, Proline; vs, versus.

**Figure 2:**
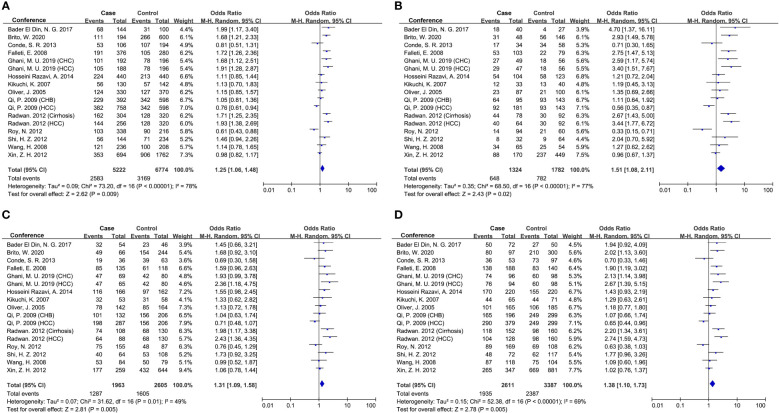
Forest plot of OR with 95% CI for associations between *TGF-β1*-509C/T polymorphism and CLD risk. **(A)** allele model, T vs C; **(B)** homozygote model, TT vs CC; **(C)** heterozygote model, CT vs CC; **(D)** dominant model, CT +TT vs CC. OR, pooled odds ratios; 95% CI, 95% confidence interval; T, Thymine; C, Cytosine.

Seventeen studies were involved in determining the association between *TGF-β1*-509C/T polymorphism and CLD risk. The sample number of the cases and control groups were 2,611 and 3,387, respectively. Except for the recessive model (TT vs CC+CT), the other four genetic models showed a significant association of *TGF-β1*-509C/T polymorphism with CLD risk (**Figure 2**; **Table 5**). The pooled OR of allele model (T vs C) was 1.25 (95% CI: 1.06-1.48, *p* = 0.009). The pooled OR of homozygote model (TT vs CC) was 1.51 (95% CI: 1.08-2.11, *p* = 0.02). The pooled OR of heterozygote model (CT vs CC) was 1.31 (95% CI: 1.09-1.58, *p* = 0.005). The pooled OR of dominant model (CT+TT vs CC) was 1.38 (95% CI: 1.10-1.73, *p* = 0.005). Taken together, these results showed that *TGF-β1*-509C/T was significantly associated with an increased risk of CLDs.

**Figure 3:**
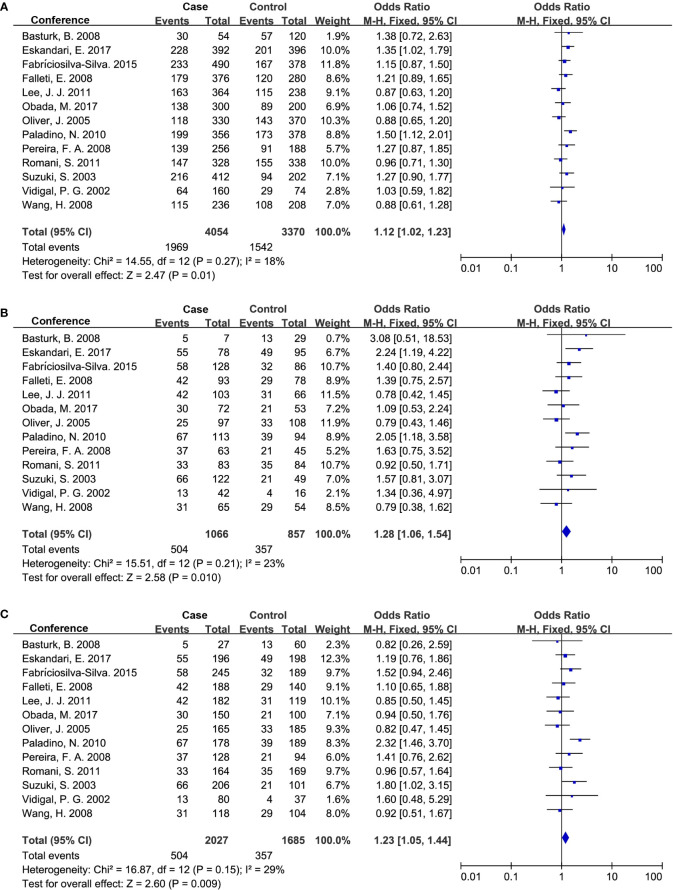
Forest plot of OR with 95% CI for associations between *TGF-β1* codon 10 polymorphism. **(A)** dominant model, Pro vs Leu) and CLD risk; **(B)** homozygote model, Pro/Pro vs Leu/Leu; **(C)** recessive model, Pro/Pro vs Leu/Leu+Leu/Pro. OR, pooled odds ratios; 95% CI, 95% confidence interval; Pro, Proline; Leu, Leucine.

Thirteen studies were involved in determining the association between *TGF-β1* codon 10 polymorphism and CLD risk. The sample number of the cases and control groups were 2,027 and 1,685, respectively. Three genetic models showed association with susceptibility to CLDs (**Figure 3**; **Table 5**), including allele model (Pro vs Leu, OR: 1.12, 95% CI: 1.02-1.23, *p* = 0.01), homozygote model (Pro/Pro vs Leu/Leu, OR: 1.28, 95% CI: 1.06-1.54, *p* = 0.01) and recessive model (Pro/Pro vs Leu/Leu+Leu/Pro, OR: 1.23, 95% CI: 1.05-1.44, *p* = 0.009). Together, our results indicated that patients with the *TGF-β1* codon 10 Pro/Pro genotype would have a higher CLD occurrence.

Nineteen studies were involved in determining the association between *TGF-β1* codon 25 polymorphism and CLD risk. The sample number of the cases and control groups were 1,896 and 1,861, respectively. In our study, *TGF-β1* codon 25 polymorphism had no significant association with the susceptibility to CLDs (**Table 5**).

### Subgroup analysis by etiologies

We conducted a subgroup analysis to assess the effect of etiology on the relationship between *TGF-β1* polymorphisms and CLD risk. As illustrated in **Table 6**, patients with the *TGF-β1*-509 TT genotype had a significantly higher risk of cirrhosis or CHC, consistent with a previous study (24). Lamentedly, *TGF-β1*-509C/T polymorphism had a limited role in HCC susceptibility. The *TGF-β1* codon 10 polymorphism was significantly associated with CHB risk (**Table 7**), which was distinct from the current literature that *TGF-β1* codon 10 polymorphism was not associated with the HBV/HCV-induced cirrhosis or HCC (19–21, 24). In addition, we concluded that the *TGF-β1* codon 25 polymorphism had a potential relationship in patients with AIH for the first time (**Table 8**).

**Table 6:** Subgroups results of the role of *TGF-β1*-509C/T polymorphism in different CLDs.

Gene model	Subgroup	Numberof study	OR (95% CI)	*P*	Test for heterogeneity		Analysis model
					*I* ^2^, %	*P_het_*	
T vs C	Overall	17	1.25 (1.06, 1.48)	0.009	78	<0.00001	RE
	HCC	5	1.28 (0.90, 1.84)	0.17	88	<0.00001	RE
	Cirrhosis	3	1.54 (1.27, 1.86)	<0.0001	42	0.18	FE
	CHB	3	1.04 (0.87, 1.23)	0.67	0	0.54	FE
	CHC	3	1.73 (1.38, 2.18)	<0.00001	0	0.85	FE
	ALD	2	0.85 (0.46, 1.57)	0.60	85	0.009	RE
	PBC	1	1.13 (0.70, 1.83)	0.62	–	–	–
TT vs CC	Overall	17	1.51 (1.08, 2.11)	0.02	77	<0.00001	RE
	HCC	5	1.57 (0.77, 3.21)	0.22	86	<0.00001	RE
	Cirrhosis	3	2.21 (1.52, 3.21)	<0.0001	35	0.22	FE
	CHB	3	1.07 (0.76, 1.51)	0.7	0	0.56	FE
	CHC	3	3.04 (1.89, 4.88)	<0.00001	0	0.72	FE
	ALD	2	0.67 (0.17, 2.71)	0.58	86	0.007	RE
	PBC	1	1.19 (0.45, 3.13)	0.73	–	–	–
CT vs CC	Overall	17	1.31 (1.09, 1.58)	0.005	49	0.01	RE
	HCC	5	1.42 (0.89, 2.25)	0.14	77	0.02	RE
	Cirrhosis	3	1.53 (1.12, 2.10)	0.008	26	0.26	FE
	CHB	3	1.19 (0.87, 1.63)	0.28	38	0.2	FE
	CHC	3	1.70 (1.15, 2.52)	0.008	0	0.86	FE
	ALD	2	0.96 (0.68, 1.35)	0.8	20	0.26	FE
	PBC	1	1.33 (0.62, 2.82)	0.46	–	–	–
CT+TT vs CC	Overall	17	1.38 (1.10, 1.73)	0.005	69	<0.00001	RE
	HCC	5	1.48 (0.86, 2.53)	0.15	85	<0.0001	RE
	Cirrhosis	3	1.74 (1.30, 2.33)	0.0002	41	0.18	FE
	CHB	3	1.15 (0.85, 1.54)	0.36	29	0.24	FE
	CHC	3	2.04 (1.41, 2.95)	0.0002	0	0.98	FE
	ALD	2	0.87 (0.47, 1.61)	0.66	72	0.06	RE
	PBC	1	1.29 (0.63, 2.61)	0.49	–	–	–
TT vs CC+CT	Overall	17	1.25 (1.00, 1.57)	0.05	65	0.0001	RE
	HCC	5	1.21 (0.80, 1.83)	0.38	73	0.005	RE
	Cirrhosis	3	1.71 (1.24, 2.36)	0.001	0	0.48	FE
	CHB	3	0.98 (0.75, 1.28)	0.87	0	0.81	FE
	CHC	3	2.11 (1.44, 3.11)	0.0001	0	0.51	FE
	ALD	2	0.70 (0.21, 2.30)	0.55	84	0.01	RE
	PBC	1	1.01 (0.42, 2.41)	0.98	–	–	–

HCC, hepatocellular carcinoma; PBC, primary biliary cirrhosis; CHB, chronic hepatitis B; CHC, chronic hepatitis C; ALD, alcoholic liver disease; OR, pooled odds ratios; 95% CI, 95% confidence interval; P, P value for Z test. I^2^, Cochrane I^2^ test; P_het_, P value for heterogeneity; RE, random-effects model; FE, fixed-effects model; C, Cytosine; T, Thymine; vs, versus.

**Table 7:** Subgroups results of the role of *TGF-β1* codon 10 polymorphism in different CLDs.

Gene model	Subgroup	Number of study	OR (95% CI)	*P*	Test for heterogeneity		Analysis model
					*I* ^2^, %	*P_het_*	
Pro vs Leu	Overall	13	1.12 (1.02, 1.23)	0.01	18	0.27	FE
	Cirrhosis	3	0.99 (0.82, 1.20)	0.92	24	0.27	FE
	CHB	2	1.35 (1.05, 1.75)	0.02	0	0.95	FE
	CHC	6	1.12 (0.97, 1.29)	0.12	0	0.84	FE
	AIH	1	1.50 (1.12, 2.01)	0.006	–	–	–
	ALD	1	0.88 (0.65, 1.20)	0.43	–	–	–
Pro/Pro vs Leu/Leu	Overall	13	1.28 (1.06, 1.54)	0.01	23	0.21	FE
	Cirrhosis	3	0.97 (0.67, 1.41)	0.87	6	0.34	FE
	CHB	2	2.33 (1.28, 4.22)	0.005	0	0.75	FE
	CHC	6	1.28 (0.96, 1.70)	0.09	0	0.84	FE
	AIH	1	2.15 (1.18, 3.58)	0.01	–	–	–
	ALD	1	0.79 (0.43, 1.46)	0.45	–	–	–
Leu/Pro vs Leu/Leu	Overall	13	1.05 (0.90, 1.23)	0.54	35	0.1	FE
	Cirrhosis	3	1.04 (0.76, 1.43)	0.81	35	0.21	FE
	CHB	2	2.58 (1.52, 4.37)	0.0004	0	0.34	FE
	CHC	6	0.95 (0.76, 1.20)	0.69	0	0.82	
	AIH	1	0.35 (0.11, 1.12)	0.08	–	–	–
	ALD	1	0.92 (0.58, 1.46)	0.72	–	–	–
Pro/Pro+Leu/Pro vs Leu/Leu	Overall	13	1.11 (0.96, 1.29)	0.15	28	0.17	FE
	Cirrhosis	3	1.02 (0.76, 1.37)	0.91	43	0.17	FE
	CHB	2	2.51 (1.50, 4.18)	0.0004	0	0.41	FE
	CHC	6	1.04 (0.84, 1.30)	0.71	0	0.92	FE
	AIH	1	1.18 (0.74, 1.86)	0.49	–	–	–
	ALD	1	0.88 (0.58, 1.35)	0.56	–	–	–
Pro/Pro vs Leu/Leu+Leu/Pro	Overall	13	1.23 (1.05, 1.44)	0.009	29	0.15	FE
	Cirrhosis	3	0.96 (0.69, 1.31)	0.78	0	0.79	FE
	CHB	2	1.13 (0.74, 1.71)	0.57	0	0.56	FE
	CHC	6	1.32 (1.04, 1.68)	0.02	0	0.54	FE
	AIH	1	2.32 (1.46, 3.70)	0.0004	–	–	–
	ALD	1	0.82 (0.47, 1.45)	0.5	–	–	–

CHB, chronic hepatitis B; CHC, chronic hepatitis C; ALD, alcoholic liver disease; AIH, autoimmune hepatitis; OR, pooled odds ratios; 95% CI, 95% confidence interval; P, P value for Z test. I^2^, Cochrane I^2^ test; P_het_, P value for heterogeneity; FE, fixed-effects model; Pro, Proline; Leu, Leucine; vs, versus.

**Table 8:** Subgroups results of the role of *TGF-β1* codon 25 polymorphism in different CLDs.

Gene model	Subgroup	Number of study	OR (95% CI)	*P*	Test for heterogeneity		Analysis model
					*I* ^2^, %	*P_het_*	
Pro vs Arg	Overall	19	1.09 (0.78, 1.52)	0.6	69	<0.00001	RE
	HCC	1	0.67 (0.30, 1.48)	0.32	–	–	–
	Cirrhosis	2	1.57 (0.95, 2.59)	0.08	0	0.42	FE
	CHB	2	1.22 (0.80, 1.87)	0.35	0	0.87	FE
	CHC	10	0.91 (0.58, 1.44)	0.68	55	0.02	RE
	AIH	2	2.37 (0.45, 12.35)	0.31	94	<0.0001	RE
	ALD	2	0.28 (0.02, 5.29)	0.40	76	0.04	RE
Pro/Pro vs Arg/Arg	Overall	14	0.97 (0.38, 2.51)	0.95	55	0.007	RE
	HCC	1	0.09 (0.00, 2.17)	0.14	–	–	–
	Cirrhosis	1	2.00 (0.15, 26.73)	0.6	–	–	–
	CHB	2	1.04 (0.28, 3.90)	0.95	38	0.2	FE
	CHC	6	0.44 (0.17, 1.14)	0.09	0	0.77	FE
	AIH	2	14.73 (3.92, 55.37)	<0.0001	0	0.48	FE
	ALD	2	0.38 (0.09, 1.63)	0.19	7	0.3	FE
Arg/Pro vs Arg/Arg	Overall	19	1.01 (0.73, 1.39)	0.98	55	0.002	RE
	HCC	1	0.69 (0.18, 2.60)	0.58	–	–	–
	Cirrhosis	2	2.15 (1.13, 4.10)	0.02	0	0.39	FE
	CHB	2	1.29 (0.80, 2.08)	0.3	0	0.49	FE
	CHC	10	0.92 (0.56, 1.51)	0.73	56	0.01	RE
	AIH	2	0.90 (0.23, 3.47)	0.88	81	0.02	RE
	ALD	2	0.33 (0.02, 4.74)	0.42	70	0.07	RE
Pro/Pro+Arg/Pro vs Arg/Arg	Overall	19	1.07 (0.76, 1.52)	0.68	64	<0.0001	RE
	HCC	1	0.60 (0.16, 2.25)	0.45	–	–	–
	Cirrhosis	2	2.14 (1.12, 4.06)	0.02	0	0.43	FE
	CHB	2	1.26 (0.80, 2.00)	0.32	0	0.78	FE
	CHC	10	0.90 (0.55, 1.50)	0.7	59	0.01	RE
	AIH	2	1.70 (0.32, 9.14)	0.54	92	0.0005	RE
	ALD	2	0.26 (0.01, 5.57)	0.39	77	0.04	RE
Pro/Pro vs Arg/Arg+Arg/Pro	Overall	14	1.00 (0.42, 2.41)	1	49	0.02	RE
	HCC	1	0.11 (0.00, 2.35)	0.16	–	–	–
	Cirrhosis	1	0.53 (0.07, 4.08)	0.54	–	–	–
	CHB	2	1.01 (0.27, 3.77)	0.99	41	0.19	FE
	CHC	6	0.53 (0.20, 1.41)	0.2	0	0.93	FE
	AIH	2	14.69 (3.98, 54.23)	<0.0001	0	0.58	FE
	ALD	2	0.51 (0.11, 2.28)	0.38	0	0.54	FE

HCC, hepatocellular carcinoma; CHB, chronic hepatitis B; CHC, chronic hepatitis C; AIH, autoimmune hepatitis; ALD, alcoholic liver disease; OR, pooled odds ratios; 95% CI, 95% confidence interval; P, P value for Z test. I^2^, Cochrane I^2^ test; P_het_, P value for heterogeneity; RE, random-effects model; FE, fixed-effects model; Pro, Proline; Arg, Arginine; vs, versus.

### Sensitivity analysis

We performed a leave-one-out sensitivity analysis to ascertain the stability of the meta-analysis by removing a single study at a time under the random-effects model. As shown in **Tables S3, S4**, the statistical significance of the result was not altered when a single study was omitted, indicating that our results were reliable and stable.

### Publication bias

**Figure 4:**
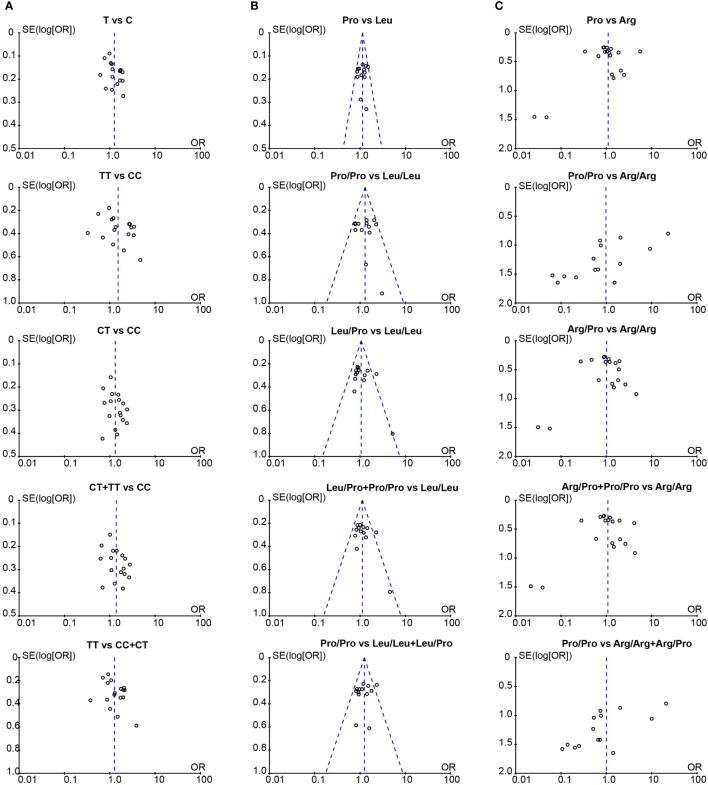
The funnel plot for publication bias assessment in the meta-analysis. **(A)** Funnel plot of *TGF-β1*-509C/T polymorphism and CLD risk; **(B)** Funnel plot of *TGF-β1* codon 10 polymorphism and CLD risk; **(C)** Funnel plot of *TGF-β1* codon 25 polymorphism and CLD risk. T, Thymine; C, Cytosine; Pro, Proline; Leu, Leucine; Arg, Arginine.

We assessed the publication bias with the Begg’s and Egger’s test, and used a funnel plot to graphically represent the bias. Our results showed there was no publication bias in studies associated with *TGF-β1*-509C/T and codon 10 (**Table 5**). Similarly, the funnel plots were approximately symmetric, indicating no evidence of potential publication bias (**Figure 4**).

### *TGF-β1* gene expression in human CLDs

**Figure 5:**
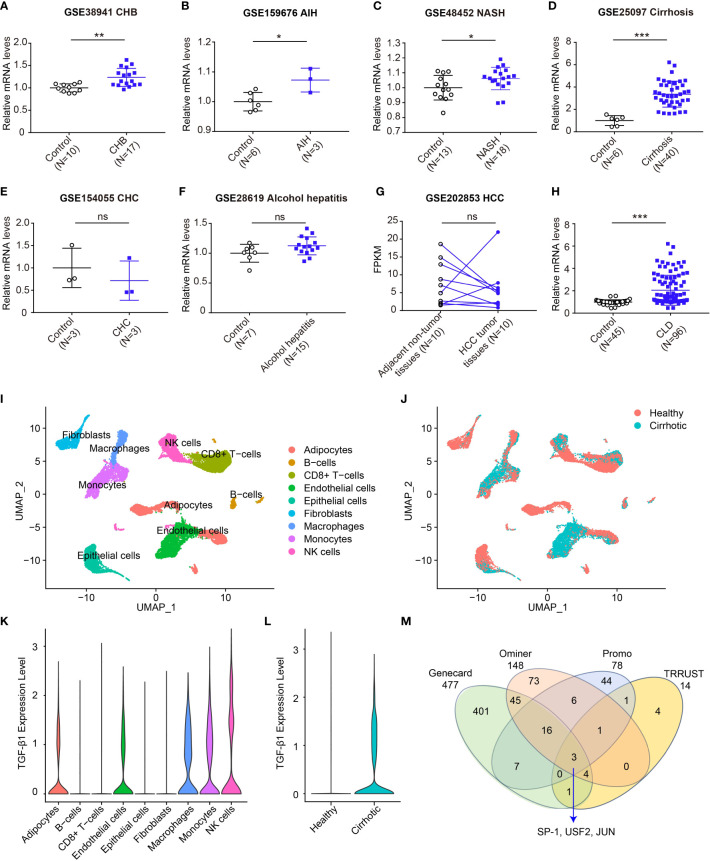
Expression of *TGF-β1* mRNA increases in CLDs. **(A-F)** Transcript levels of TGF-β1 in healthy individuals or patients with CHB (**A**, GSE38941), AIH (**B**, GSE159676), NASH (**C**, GSE48452), cirrhosis (**D**, GSE25097), CHC (**E**, GSE154055), and alcohol hepatitis (**F**, GSE28619); **(G)** Transcript levels of *TGF-β1* in adjacent non-tumor or HCC tumor (GSE202853); **(H)** Transcript levels of *TGF-β1* in healthy individuals or patients with CLDs; **(I, J)** UMAP plot showing all liver non-parenchymal cells dependent on the cluster **(I)** and dependent on the origin **(J)**; **(K)** TGF-β1 gene expression in all cell clusters; **(L)** TGF-β1 gene expression in healthy individuals and liver cirrhosis patients; **(M)** The transcription factor analysis of TGF-β1. Data are mean ± SD. *P<0.05, **P<0.01, and ***P<0.001 vs. Control. ns, not significant. Significance was calculated by two-tailed unpaired Student’s *t*-test.

Studies have shown that *TGF-β1*-509C/T and codon 10 displayed increased expression and plasma concentration of TGF-β1 (21, 31–33), implying that *TGF-β1* genetic variants could affect TGF-β1 protein expression. Here, the association of *TGF-β1* polymorphisms with the gene expression of TGF-β1 was explored. We firstly evaluated the *TGF-β1* mRNA levels in all sorts of CLD patients based on GEO datasets (**Figure 5**). Compared to healthy controls, a significantly higher mRNA level of *TGF-β1* was observed in patients with CHB, AIH, NASH and cirrhosis (**Figures 5A-D**). Nevertheless, there were no significant differences in *TGF-β1* expression between control and patients with CHC, alcohol hepatitis, and HCC (**Figures 5E-G**). Furthermore, we integrated all data and compared the difference in *TGF-β1* mRNA levels. Results showed that the gene expression of *TGF-β1* was markedly higher in CLD patients compared to healthy controls (**Figure 5H**).

Among these etiologies of CLDs, the *TGF-β1* mRNA level was the most significantly associated with cirrhosis (**Figure 5D**). We further explored the *TGF-β1* expression patterns in a published scRNA-seq dataset of human liver non-parenchymal cells from patients with cirrhosis and healthy individuals (37). Unsupervised clustering using UMAP uncovered nine cell lineages containing cells from both healthy and cirrhotic livers (**Figures 5I, J**). *TGF-β1* was mainly expressed in natural killer (NK) cells, macrophages, monocytes, adipocytes and endothelial cells (**Figure 5K**). Notably, cirrhotic patients had a significantly elevated expression of *TGF-β1* (**Figure 5L**). Above all, these data further demonstrated a remarkable association between *TGF-β1* gene expression and CLDs.

### Transcription analysis of TGF-β1

*TGF-β1*-509C/T and -800G/A are located in the promoter region at positions -509 and -800, which might result in a transcript shift of *TGF-β1* by affecting the activity of the promoter. Transcription factors bind DNA in a sequence-specific way and play an important role in regulating gene transcription. Thus, we wonder if the *TGF-β1* genetic variants interfere with the DNA binding of its transcription factors. We combined the candidates that were reported in Genecard, Ominer, Promo, and TRRUST databases to identify the most potential transcription factors involved in *TGF-β1* transcription. Based on the overlapping data, we eventually identified three transcription factors, including specific protein-1 (SP-1), upstream stimulatory factor 2 (USF2), and JUN (**Figure 5M**).

SP-1 is the main nuclear protein involved in the regulation of *TGF-β1* gene activation, which can greatly boost the activity of the *TGF-β1* promoter (44–47). There are 11 conserved sequences of SP-1 binding sites (5’-GGGCGG) in the human *TGF-β1* promoter. According to the present findings, *TGF-β1*-509C/T and -800G/A genetic variants weren’t involved in the binding sites of SP-1 in the *TGF-β1* promoter, revealing these two variants may not affect the transcriptional regulation of SP-1 on *TGF-β1* gene.

USF2 belongs to the basic helix-loop-helix leucine zipper family of transcription factors characterized by a highly conserved COOH-terminal domain responsible for dimerization and DNA binding (48, 49). Studies found that high glucose concentrations could induce USF2 binding to the *TGF-β1* promoter region -1013/-1002, enhancing *TGF-β1* promoter activity (50, 51). In addition, two specific binding sites for USF were identified in the *TGF-β1* promoter: -1,846 approximately -1,841 (CACATG) and -621 approximately -616 (CATGTG) (52), which did not include the positions -509 and -800. Thus, the current study provides no evidence that *TGF-β1*-509C/T and -800G/A could influence the transcriptional regulation of USF2 on the *TGF-β1* gene.

JUN is a transcription factor that recognizes and binds to the activator protein-1 (AP-1) consensus motif (53). Studies have validated that TGF-β1 could induce transcription of JUN mRNA through SMAD7 dependent/independent feedback manner (54, 55). Moreover, one study found that JUN could be involved in the promoter of *TGF-β1*, while its role and binding site in the *TGF-β1* promoter were unknown (56). More investigations should be taken to uncover the binding site of JUN in the *TGF-β1* promoter, then to determine if the binding sites covered the position of *TGF-β1*-509C/T and -800G/A genetic variants, and finally to figure out the underlying mechanism by which *TGF-β1* polymorphism regulated the binding activity between transcription factors and *TGF-β1* promoter to affect *TGF-β1* transcription.

### Structural analysis of TGF-β1 protein

**Figure 6:**
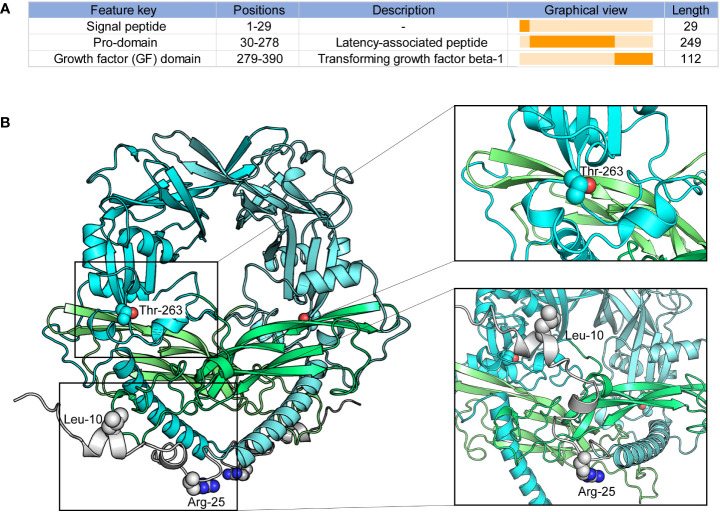
Peptide features and structure of TGF-β1. **(A)** Peptide features of TGF-β1; **(B)** Overall structure of TGF-β1. Protein structures and residues are shown in cartoon and sphere representation, respectively.

The *TGF-β1* codon 10 (CTG > CCG), codon 25 (CGG > CCG), and codon 263 (ACC > ATC) variants lead to amino-acid substitutions Leu10Pro, Arg25Pro, and Thr263Ile (**Table S5**). We investigated these codon variants based on the TGF-β1 crystal structure. TGF-β1 encodes a polypeptide comprising a signal peptide with 29 residues, a 249-residue pro-domain (latency-associated peptide), and a 112-residue growth factor (GF) domain (**Figure 6A**) (57, 58). And TGF-β1 is covalently linked to form dimers in the endoplasmic reticulum (58, 59). We modeled the overall crystal structure of TGF-β1 using the GalaxyHomomer method of GalaxyWeb (39, 40) and selected the best-predicted model for further analysis.

As illustrated in **Figure 6B**, *TGF-β1* codon 10 and codon 25 were located in the signal peptide of TGF-β1, which domain hasn’t been confirmed based on experiments. We could not conclude any more results on how *TGF-β1* codon 10 and codon 25 variants affect the 3D structure of TGF-β1. Our results showed that the *TGF-β1* codon 263 was located in the pro-domain, close to the region where the TGF-β1 dimerization interacts, implying that the *TGF-β1* codon 263 variant may alter the dimerization of TGF-β1. Latent TGF-β1 is covalently linked to form disulfide dimers and binds to the latent TGF-β binding protein (LTBP) (57–59). Once TGF-β1 was released from the LTBP, TGF-β1 could be secreted and interact with membrane receptors to initiate signal transduction (58, 60). Therefore, the *TGF-β1* codon 263 variant might affect the binding of TGF-β1 to LTBP by altering the dimerization of TGF-β1, thus affecting TGF-β1 secretion and signal transduction.

## Discussion

In the present study, we pooled 35 studies and conducted a meta-analysis to evaluate the frequency of *TGF-β1* SNPs in CLD patients and healthy individuals. To our knowledge, this is the first systematic review and meta-analysis to comprehensively assess all *TGF-β1* SNPs associated with CLDs. Our results revealed that the *TGF-β1*-509C/T and codon 10 were significantly associated with CLD risk. Individuals with the *TGF-β1*-509 (TT or CT allele) or codon 10 (Pro/Pro allele) showed an increased risk of CLDs in the overall population. In particular, we identified that *TGF-β1*-509C/T was strongly associated with patients with cirrhosis or CHC, while *TGF-β1* codon 10 was more associated with patients with CHB. *TGF-β1* codon 25 exhibited a relationship with AIH risk. Moreover, we identified that the mRNA level of *TGF-β1* was significantly higher in a variety of CLDs, and explored the potential mechanisms by which *TGF-β1* polymorphisms influence CLD risk based on transcription factors and protein structure of TGF-β1.

CLDs are extremely common clinical conditions caused by diverse etiologies, such as CHB, CHC, AIH, cirrhosis, NASH, ALD, PBC and HCC, affecting the health of millions of people (1, 2). When the inflammatory balance is disrupted in the liver, sustained inflammation responses can lead to the occurrence of CLDs. TGF-β1 encoded by *TGF-β1* is a multifunctional immune regulator that contributes to various immunologic processes in CLDs (9). TGF-β1 not only shows the anti-inflammatory and immune surveillance properties in HCC but could also promote T cell differentiation and activation in CHB, CHC and NASH (11–15). Studies have indicated that *TGF-β1* genetic variants could trigger the susceptibility of CLDs, acting as a potential candidate for predicting CLD risk. Up to now, a few studies have investigated the relationship between *TGF-β1*-509C/T and codon 10 polymorphisms and the risk of HBV/HCV-induced cirrhosis and HCC. However, these results were inconsistent and controversial (**Table S6**). Some studies showed that the *TGF-β1*-509 TT genotype showed an association with HCV-induced cirrhosis and HCC (20, 23, 24), and the *TGF-β1* codon 10 Pro/Pro genotype had a slight effect on HCC risk (20). But more studies just did not detect a significant association between *TGF-β1* polymorphisms and cirrhosis or HCC susceptibility (19, 21, 22, 25). As a whole, there was no systematic study to evaluate the association of *TGF-β1* polymorphisms in CLDs. Our study consolidated all available data with *TGF-β1* polymorphisms associated with diverse etiologies of CLDs and identified that *TGF-β1*-509C/T and codon 10 polymorphisms were highly associated with an increased risk of CLDs for the first time. *TGF-β1*-509C/T was significantly associated with CLD susceptibility under all genetic models except the recessive model (TT vs CC+CT; **Table 5**). For *TGF-β1* codon 10, the fixed-effect meta-analysis showed that the Pro/Pro genotype conferred a significantly increased risk to CLDs under the allele contrast model (pooled OR = 1.28, 95% CI: 1.06-1.54; **Table 5**). Lamentedly, the *TGF-β1* codon 25 showed no relationship with LCD risk (**Table 5**).

In the subgroup analysis based on etiologies, we found that *TGF-β1*-509C/T had a statistical association with cirrhosis and CHC under all genetic models (**Table 6**), consistently with the previous study (24). The *TGF-β1* codon 10 was significantly associated with CHB under allele model (pooled OR = 1.35, 95% CI: 1.05-1.75), homozygote model (pooled OR = 2.33, 95% CI: 1.28–4.22), heterozygote model (pooled OR = 2.58, 95% CI: 1.52-4.37) and dominant model (pooled OR = 2.51, 95% CI: 1.50-4.18) (**Table 7**). Interestingly, *TGF-β1* codon 25 Pro/Pro genotype showed a statistically significant association with AIH risk under homozygote model (pooled OR = 14.73, 95% CI: 3.92-55.37) and recessive model (pooled OR = 14.69, 95% CI: 3.98-54.23) (**Table 8**). Taken together, our data demonstrated that *TGF-β1*-509C/T and codon 10 polymorphisms could be feasible to screen for individuals at risk for CLDs, especially for cirrhosis, CHB, and CHC.

Studies have observed that *TGF-β1*-509C/T and codon 10 polymorphisms were associated with HCC risk (20). However, we didn’t reveal similar results based on the involved 35 eligible studies (**Tables 6** and **7**). To get clear on which *TGF-β1* polymorphism could affect HCC risk, we analyzed the *TGF-β1* variants on the cBioPortal database. Our results showed that the most common variants of *TGF-β1* in HCC were missense mutations in G29E, A105S, D191N, and F321L, which might be the more precise genetic factors of *TGF-β1* to influence HCC susceptibility (**Figure S1**).

The fact that CLDs have different pathogenic and pathogenesis and are regulated by various genes. Based on our findings that *TGF-β1* polymorphisms contribute differently to CLD susceptibility, we hypothesized that *TGF-β1* polymorphism might be associated with CLD-related genes and play distinct regulatory roles. Lamentedly, the relationship between *TGF-β1* polymorphism and CLD-related genes has not been identified, except the different interactions between TGF-β1 signaling and CLD-related genes, such as *Ki67* and *P53*, well-known HCC-related genes (61). A positive correlation between the activated TGF-β1 signaling pathway and high Ki67 expression due to the abnormal proliferation of cancer cells in tumor tissues (62). *P53* mutants affected the transcriptional activation of TGF-β (63, 64). Overall, the interactions between *TGF-β1* polymorphism and different CLD-related genes are interesting and undiscovered, which is worth further studies.

To reduce sampling bias in case-control studies, studies in which *TGF-β1* SNPs demonstrated a departure from HWE in controls were excluded. Furthermore, sensitivity analysis was also applied to evaluate the potential source of heterogeneity of *TGF-β1*-509C/T and codon 25 (**Tables S3, S4**). After removing a single study at a time under the random-effects model, the positive association between *TGF-β1*-509C/T and the CLD risk was not changed (**Table S3**). In the current study, we did not observe any publication bias in *TGF-β1*-509C/T and codon 10 (**Table 5**; **Figure 4**), confirming our findings that *TGF-β1*-509C/T and codon 10 were positively associated with CLD risk.

Studies have shown that the mRNA and protein levels of TGF-β1 were significantly upregulated in patients with cirrhosis (26, 27), and TGF-β1 expression was higher in tumor tissues of HCC (28–30). Clinic studies revealed that *TGF-β1* gene variants could affect TGF-β1 levels in individuals. For instance, individuals with *TGF-β1*-509C/T or codon 10 showed an increased level of TGF-β1 expression (31–33). However, the underlying mechanism is still unclear. Accordingly, we comprehensively analyzed the *TGF-β1* mRNA levels in different etiologies of CLDs and observed that *TGF-β1* was higher in patients with CHB, AIH, NASH and cirrhosis, compared to healthy controls (**Figures 5A-D**). Moreover, we integrated the different types of CLDs and validated that *TGF-β1* was significantly upregulated in CLD patients (**Figure 5H**). Collectively, these results suggested that high *TGF-β1* expression may be a promising biomarker for CLD diagnosis.

The *TGF-β1*-509C/T and -800G/A are located in the promoter region at positions -509 and -800, separately, which might change the transcription of *TGF-β1* by affecting the binding between transcription factors and *TGF-β1*. We explored the potential transcription factors of *TGF-β1* based on publicly available databases and observed three crucial transcription factors of *TGF-β1* (**Figure 5M**). Studies have reported the binding sites of SP-1 and USF2 in the *TGF-β1* promoter, which did not cover the position of *TGF-β1*-509C/T and -800G/A genetic variants. Based on the current results, transcriptional regulation of SP-1 or USF2 in *TGF-β1* did not affect *TGF-β1* expression associated with *TGF-β1*-509C/T and -800G/A. On the other hand, studies have found that JUN was occupied in the *TGF-β1* promoter, but the binding sites haven’t been reported (56). Guess that if we could figure out the vital transcription factors of *TGF-β1* with the binding sites covering -509 or -800 in the *TGF-β1* promoter region, we might uncover the underlying mechanisms by which *TGF-β1*-509C/T and -800G/A affect TGF-β1 levels in patients with CLDs. Nevertheless, our findings could provide an idea of how *TGF-β1* genetic variants in the promoter affect *TGF-β1* expression.

In the endoplasmic reticulum, the pro-domain and GF domain of latent TGF-β1 are covalently linked to form disulfide dimers (57–59). At the same time, the pro-domain binds to the potential TGF-β binding protein to form disulfide connections. And after being released from its latent form, TGF-β1 is secreted and directly interacts with membrane receptors to initiate signal transduction (58, 60). *TGF-β1* codon 10, codon 25, and codon 263 change the amino-acid substitutions, possibly leading to changes in TGF-β1 expression and secretion. We investigated the potential effects of *TGF-β1* codon variants based on the crystal structure of the TGF-β1 protein. The 1-29 of the TGF-β1 domain as a signal peptide hasn’t been solved based on experiments. Thus, we can’t conclude any useful results for *TGF-β1* codon 10 and codon 25 variants. As shown in **Figure 6**, codon 263 was located close to where the dimerization of TGF-β1 interacts. Consequently, we had a hypothesis that the *TGF-β1* codon 263 variant may affect the dimerization of TGF-β1 in the endoplasmic reticulum and then change the TGF-β1 secretion, which remains further defined.

## Conclusion

**Figure 7:**
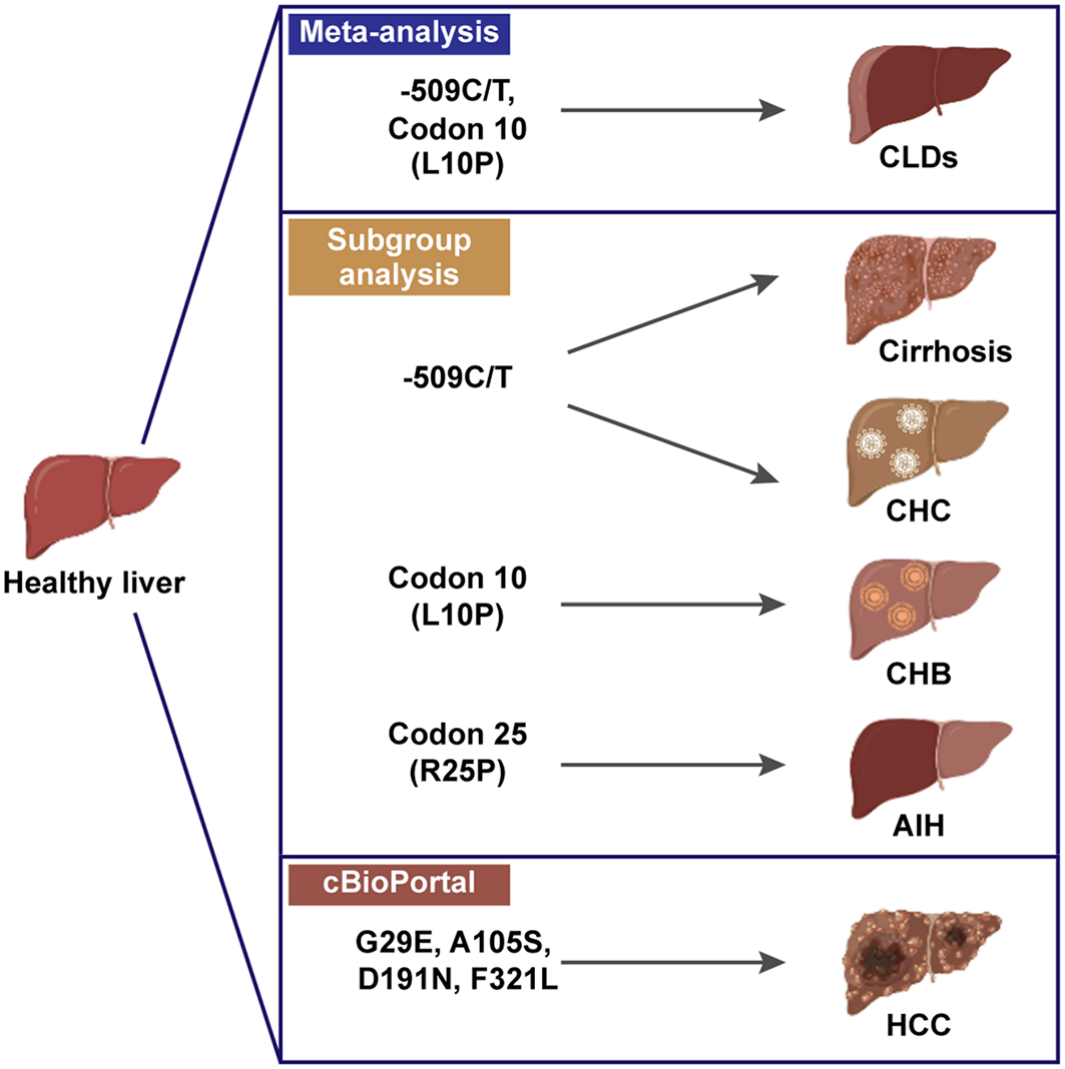
Schematic of *TGF-β1* polymorphisms in susceptibility of CLDs, created in BioRender.com.

Our analysis provides evidence supporting *TGF-β1*-509C/T (rs1800469) and *TGF-β1* codon 10 (rs1800470) as susceptibility factors for CLD occurrence for the first time (**Figure 7**). *TGF-β1*-509 TT genotype and T allele were correlated with increased CLD risk, specifically with cirrhosis and CHC-induced CLD individuals. The *TGF-β1* codon 10 polymorphism was also correlated with increased CLD susceptibility, playing a more significant role in predicting the occurrence of CHB. Our exploration of the underlying mechanisms by which *TGF-β1* polymorphisms affect CLD risk by regulating TGF-β1 expression would provide a better understanding of the association between the immune regulator and CLD pathogenesis. Notwithstanding the significant findings obtained from the current study, several limitations should still be considered. First, the pathogenesis of CLDs is sophisticated and engages potential interactions between genes and the environment. More studies with sufficient statistics are required for a more thorough assessment. Second, except for etiology, *TGF-β1* polymorphisms on CLD susceptibility could be affected by ethnicity, sex, age, etc. We did not carry out a subgroup analysis to assess these effects on the association between *TGF-β1* polymorphisms and CLD risk. Third, the sample size of our study was relatively small, and we had two studies with NOS scores of 6, which were of intermediate quality. Finally, we did not perform a meta-analysis on *TGF-β1*-800G/A and codon 263 for the small sample size of aggregate analysis. Further investigation should be carried out to verify this relationship and explore other aspects of the CLD risk.

## Data availability statement

The original contributions presented in the study are included in the article/**Supplementary Material**. Further inquiries can be directed to the corresponding authors.

## Author contributions

Conceptualization, QW, JW, QH, BY, and XC. methodology, XC, HZ, ZY and YD. software, XC and ZY. validation, HZ, ZY, YD and XD. investigation, XC and ZY. data curation, JW, XC and ZY. writing—original draft preparation, QW, JW and XC. writing—review and editing, QW, JW and XC. All authors contributed to the article and approved the submitted version.

## Funding

This research was funded by the Zhejiang Provincial Natural Science Foundation (No. LY22H310001, No. LR21H310001) and the Fundamental Research Funds for the Central Universities.

## Conflict of interest

The authors declare that the research was conducted in the absence of any commercial or financial relationships that could be construed as a potential conflict of interest.

## Publisher’s note

All claims expressed in this article are solely those of the authors and do not necessarily represent those of their affiliated organizations, or those of the publisher, the editors and the reviewers. Any product that may be evaluated in this article, or claim that may be made by its manufacturer, is not guaranteed or endorsed by the publisher.
