# Supplementary material for: Genetic dominance of transforming growth factor-β1 polymorphisms in chronic liver disease
Source: Front Immunol. 2022 Nov 16;13:1058532. doi: 10.3389/fimmu.2022.1058532 (PMC9708878; doi:10.3389/fimmu.2022.1058532)
Supplement: Supplementary file 1 [file DataSheet_1.docx]

Supplementary Material

# Supplementary Figures and Tables

Supplementary Table 1. The genotype and allele frequencies of TGF-β1-800G/A distribution of included studies.

Supplementary Table 2. The genotype and allele frequencies of TGF-β1 codon 263 distribution of included studies.

Supplementary Table 3. Sensitivity analysis of TGF-β1-509C/T polymorphism in different CLDs.

Supplementary Table 4. Sensitivity analysis of TGF-β1 codon 25 polymorphism in different CLDs.

Supplementary Table 5. The features of TGF-β1 SNPs.

Supplementary Table 6. Association of TGF-β1 Polymorphisms with HCC and cirrhosis in published meta-analysis.

Supplementary Figure 1. TGF-β1 gene mutations in patients with HCC.

**Supplementary Table 1. The genotype and allele frequencies of *TGF-β1*-800G/A distribution of included studies.**

| Reference | Disease | Number | | Case | | | Control | | | *P* for HWE | |
| --- | --- | --- | --- | --- | --- | --- | --- | --- | --- | --- | --- |
|  |  | Case/n | Control/n | GG | GA | AA | GG | GA | AA | Case | Control |
| Dondeti, M. F. et al. (2017) | CHB | 115 | 119 | 90 | 19 | 6 | 97 | 17 | 5 | 0.002 | 0.002 |
| Falleti, E. et al. (2008) | Cirrhosis | 188 | 140 | 166 | 22 | 0 | 116 | 24 | 0 | 0.394 | 0.267 |
| Kikuchi, K. et al. (2007) | PBC | 65 | 71 | 65 | 0 | 0 | 71 | 0 | 0 | / | / |

Abbreviations: CHB, chronic hepatitis B; PBC, primary biliary cirrhosis; HWE, Hardy–Weinberg Equilibrium; G, Guanine; A, Adenine.

**Supplementary Table 2. The genotype and allele frequencies of *TGF-β1* codon 263 distribution of included studies.**

| Reference | Disease | Number | | Case | | | Control | | | *P* for HWE | |
| --- | --- | --- | --- | --- | --- | --- | --- | --- | --- | --- | --- |
|  |  | Case/n | Control/n | CC | CT | TT | CC | CT | TT | Case | Control |
| Dondeti, M. F. et al. (2017) | CHB | 115 | 119 | 11 | 85 | 19 | 35 | 75 | 9 | 0.000 | 0.000 |

Abbreviations: CHB, chronic hepatitis B; HWE, Hardy–Weinberg Equilibrium; C, Cytosine; T, Thymine.

**Supplementary Table 3. Sensitivity analysis of *TGF-β1*-509C/T polymorphism in different CLDs.**

| SNPs | Reference | OR (95% CI) | | | | |
| --- | --- | --- | --- | --- | --- | --- |
|  |  | T vs C | TT vs CC | CT vs CC | CT+TT vs CC | TT vs CC+CT |
| -509C/T | Bader El Din, N. G. 2017 | 1.22 (1.03, 1.45) | 1.44 (1.03, 2.02) | 1.31 (1.07, 1.59) | 1.36 (1.07, 1.71) | 1.21 (0.97, 1.52) |
|  | Brito, W. 2020 | 1.23 (1.03, 1.46) | 1.45 (1.03, 2.03) | 1.29 (1.06, 1.57) | 1.35 (1.07, 1.70) | 1.21 (0.96, 1.52) |
|  | Conde, S. R. 2013 | 1.28 (1.08, 1.52) | 1.58 (1.12, 2.23) | 1.34 (1.11, 1.62) | 1.42 (1.13, 1.79) | 1.28 (1.01, 1.62) |
|  | Falleti, E. 2008 | 1.22 (1.03, 1.45) | 1.45 (1.03, 2.04) | 1.29 (1.06, 1.58) | 1.35 (1.07, 1.71) | 1.21 (0.96, 1.52) |
|  | Ghani, M. U. 2019 (CHC) | 1.23 (1.04, 1.46) | 1.46 (1.04, 2.07) | 1.28 (1.06, 1.56) | 1.35 (1.07, 1.70) | 1.23 (0.98, 1.56) |
|  | Ghani, M. U. 2019 (HCC) | 1.22 (1.03, 1.44) | 1.44 (1.03, 2.02) | 1.27 (1.05, 1.53) | 1.33 (1.06, 1.67) | 1.22 (0.97, 1.54) |
|  | Hosseini Razavi, A. 2014 | 1.26 (1.05, 1.51) | 1.54 (1.07, 2.21) | 1.29 (1.06, 1.58) | 1.38 (1.08, 1.76) | 1.29 (1.01, 1.64) |
|  | Kikuchi, K. 2007 | 1.26 (1.06, 1.50) | 1.53 (1.08, 2.17) | 1.31 (1.08, 1.59) | 1.38 (1.09, 1.75) | 1.27 (1.00, 1.60) |
|  | Oliver, J. 2005 | 1.26 (1.05, 1.51) | 1.53 (1.07, 2.17) | 1.33 (1.08, 1.62) | 1.40 (1.10, 1.78) | 1.26 (0.99, 1.59) |
|  | Qi, P. 2009 (CHB) | 1.27 (1.06, 1.52) | 1.55 (1.08, 2.22) | 1.33 (1.09, 1.62) | 1.40 (1.10, 1.79) | 1.28 (1.00, 1.63) |
|  | Qi, P. 2009 (HCC) | 1.30 (1.10, 1.53) | 1.63 (1.18, 2.24) | 1.37 (1.15, 1.63) | 1.45 (1.17, 1.80) | 1.32 (1.05, 1.65) |
|  | Radwan. 2012 (Cirrhosis) | 1.22 (1.03, 1.45) | 1.45 (1.03, 2.05) | 1.27 (1.05, 1.54) | 1.34 (1.06, 1.68) | 1.23 (0.97, 1.55) |
|  | Radwan. 2012 (HCC) | 1.22 (1.03, 1.44) | 1.43 (1.02, 1.99) | 1.26 (1.05, 1.51) | 1.32 (1.06, 1.64) | 1.22 (0.97, 1.53) |
|  | Roy, N. 2012 | 1.30 (1.11, 1.53) | 1.65 (1.20, 2.26) | 1.35 (1.12, 1.64) | 1.45 (1.16, 1.81) | 1.32 (1.07, 1.63) |
|  | Shi, H. Z. 2012 | 1.24 (1.04, 1.48) | 1.49 (1.06, 2.11) | 1.29 (1.06, 1.57) | 1.36 (1.07, 1.72) | 1.25 (0.99, 1.58) |
|  | Wang, H. 2008 | 1.26 (1.05, 1.50) | 1.53 (1.08, 2.18) | 1.33 (1.09, 1.62) | 1.40 (1.10, 1.78) | 1.26 (0.99, 1.59) |
|  | Xin, Z. H. 2012 | 1.28 (1.06, 1.53) | 1.57 (1.09, 2.26) | 1.34 (1.09, 1.64) | 1.42 (1.11, 1.81) | 1.29 (1.01, 1.66) |

Abbreviations: SNPs, single-nucleotide polymorphisms; OR, pooled odds ratios; 95% CI, 95% confidence interval; C, Cytosine; T, Thymine; vs, versus.

**Supplementary Table 4. Sensitivity analysis of *TGF-β1* codon 25 polymorphism in different CLDs.**

| SNPs | Reference | OR (95% CI) | | | | |
| --- | --- | --- | --- | --- | --- | --- |
|  |  | Pro vs Arg | Pro/Pro vs Arg/Arg | Arg/Pro vs Arg/Arg | Pro/Pro+Arg/Pro vs Arg/Arg | Pro/Pro vs Arg/Arg+Arg/Pro |
| Codon 25 | Armendáriz. 2008 (ALD) | 1.13 (0.82, 1.56) | 1.11 (0.43, 2.91) | 1.03 (0.75, 1.42) | 1.12 (0.80, 1.56) | 1.08 (0.44, 2.68) |
|  | Armendáriz. 2008 (CHC) | 1.14 (0.83, 1.57) | 1.17 (0.46, 2.98) | 1.04 (0.76, 1.41) | 1.12 (0.81, 1.56) | 1.13 (0.47, 2.76) |
|  | Dondeti, M. F. 2017 | 1.08 (0.76, 1.55) | 1.06 (0.40, 2.83) | 0.97 (0.69, 1.37) | 1.06 (0.73, 1.53) | 1.10 (0.45, 2.70) |
|  | Fabríciosilva-Silva. 2015 | 1.11 (0.77, 1.59) | 0.97 (0.35, 2.73) | 1.02 (0.71, 1.46) | 1.09 (0.75, 1.60) | 1.01 (0.39, 2.61) |
|  | Falleti, E. 2008 | 1.05 (0.74, 1.49) | - | 0.95 (0.68, 1.33) | 1.03 (0.72, 1.47) | - |
|  | Hosseini Razavi, A. 2014 | 1.08 (0.75, 1.54) | 0.88 (0.31, 2.50) | 1.00 (0.70, 1.42) | 1.06 (0.73, 1.55) | 0.91 (0.34, 2.39) |
|  | Maria. 2013 | 1.08 (0.77, 1.53) | - | 0.99 (0.71, 1.39) | 1.07 (0.75, 1.52) | - |
|  | Nomair. 2021 (Cirrhosis) | 1.08 (0.76, 1.54) | 0.91 (0.33, 2.50) | 0.97 (0.70, 1.33) | 1.04 (0.73, 1.47) | 1.05 (0.41, 2.68) |
|  | Nomair. 2021 (HCC) | 1.13 (0.80, 1.59) | 1.13 (0.44, 2.91) | 1.02 (0.73, 1.43) | 1.10 (0.77, 1.57) | 1.15 (0.48, 2.76) |
|  | Obada, M. 2017 | 1.10 (0.77, 1.57) | 0.98 (0.36, 2.69) | 1.01 (0.71, 1.43) | 1.08 (0.75, 1.57) | 1.01 (0.40, 2.57) |
|  | Oliver, J. 2005 | 1.11 (0.77, 1.59) | 0.98 (0.35, 2.76) | 1.02 (0.71, 1.45) | 1.09 (0.75, 1.59) | 1.01 (0.39, 2.63) |
|  | Paladino, N. 2010 | 1.09 (0.76, 1.57) | 0.80 (0.30, 2.12) | 1.07 (0.77, 1.49) | 1.11 (0.76, 1.61) | 0.83 (0.35, 2.00) |
|  | Pereira, F. A. 2008 | 1.20 (0.88, 1.63) | 0.99 (0.36, 2.71) | 1.11 (0.83, 1.47) | 1.19 (0.87, 1.63) | 1.01 (0.40, 2.56) |
|  | Romani, S. 2011 | 1.09 (0.77, 1.56) | 1.01 (0.37, 2.76) | 0.99 (0.70, 1.41) | 1.07 (0.74, 1.56) | 1.04 (0.41, 2.64) |
|  | Sánchez-Parada. 2013 | 1.08 (0.77, 1.53) | - | 0.99 (0.71, 1.39) | 1.07 (0.75, 1.52) | - |
|  | Vidigal, P. G. 2002 | 1.06 (0.76, 1.50) | 0.93 (0.34, 2.54) | 0.98 (0.70, 1.37) | 1.05 (0.73, 1.49) | 0.97 (0.38, 2.45) |
|  | Yousefi, A. 2019 | 0.98 (0.75, 1.27) | 0.78 (0.37, 1.62) | 0.97 (0.69, 1.35) | 0.98 (0.71, 1.34) | 0.81 (0.42, 1.55) |
|  | Zein. 2004 (Caucasian) | 1.06 (0.76, 1.49) | - | 0.97 (0.70, 1.35) | 1.04 (0.73, 1.48) | - |
|  | Zein. 2004 (Egyptian) | 1.08 (0.77, 1.52) | - | 0.99 (0.71, 1.39) | 1.06 (0.74, 1.52) | - |

Abbreviations: SNPs, single-nucleotide polymorphisms; OR, pooled odds ratios; 95% CI, 95% confidence interval; Pro, Proline; Arg, Arginine; vs, versus.

**Supplementary Table 5. The features of *TGF-β1* SNPs.**

| SNP | Position | Consequence | Variation Type | Amino acid [Codon] |
| --- | --- | --- | --- | --- |
| (*TGF-β1*-800G/A) rs1800468 | chr19:41354682 (GRCh38.p13) | 2KB Upstream Transcript Variant | SNV | N/A |
| (*TGF-β1*-509C/T) rs1800469 | chr19:41354391 (GRCh38.p13) | 2KB Upstream Transcript Variant | SNV | N/A |
| (*TGF-β1* codon 10) rs1800470 | chr19:41353016 (GRCh38.p13) | Missense Variant | SNV | L [CTG]> P [CCG] |
| (*TGF-β1* codon 25) rs1800471 | chr19:41352971 (GRCh38.p13) | Missense Variant | SNV | R [CGG] > P [CCG] |
| (*TGF-β1* codon 263)  rs1800472 | chr19:41341955 (GRCh38.p13) | Missense Variant | SNV | T [ACC] > I [ATC] |

Abbreviations: SNPs, single-nucleotide polymorphisms; SNV, single nucleotide variation; N/A, not applicable; L, Leucine; P, Proline; R, Arginine; T, Threonine; I, Isole

**Supplementary Table 6. Association of *TGF-β1* Polymorphisms with HCC and cirrhosis in published meta-analysis.**

| DOI | Reference | Disease | Correlation | |
| --- | --- | --- | --- | --- |
|  |  |  | -509C/T | Codon 10 |
| 10.1111/j.1872-034X.2011.00958.x | Xiang,T X. et al (2012) | HCC | no | no |
| 10.1089/gtmb.2013.0268 | Guo, Y. et al (2013) | HCC | yes | yes |
| 10.1007/s11033-012-2090-1 | Wu, X D. et al (2013) | Cirrhosis | no | no |
| 10.18632/oncotarget.13218 | Lu, W Q. et al (2016) | Cirrhosis | no | - |
| 10.4149/neo_2016_615 | N. TOSHIKUNI. et al (2016) | HBV-HCC | no | - |
|  |  | HCV-HCC | yes | - |
| 10.1016/j.meegid.2017.12.019 | Guo, P F. et al (2017) | HBV-Cirrhosis | no | no |
|  |  | HCV-Cirrhosis | yes | no |
| 10.1016/j.gene.2020.144365 | Zhang, C. et al (2020) | HCC risk in Asian populations | no | - |

Abbreviations: DOI, Digital Object Unique Identifier; HCC, hepatocellular carcinoma; HBV, hepatitis B virus; HCV, hepatitis C virus; C, Cytosine; T, Thymine.


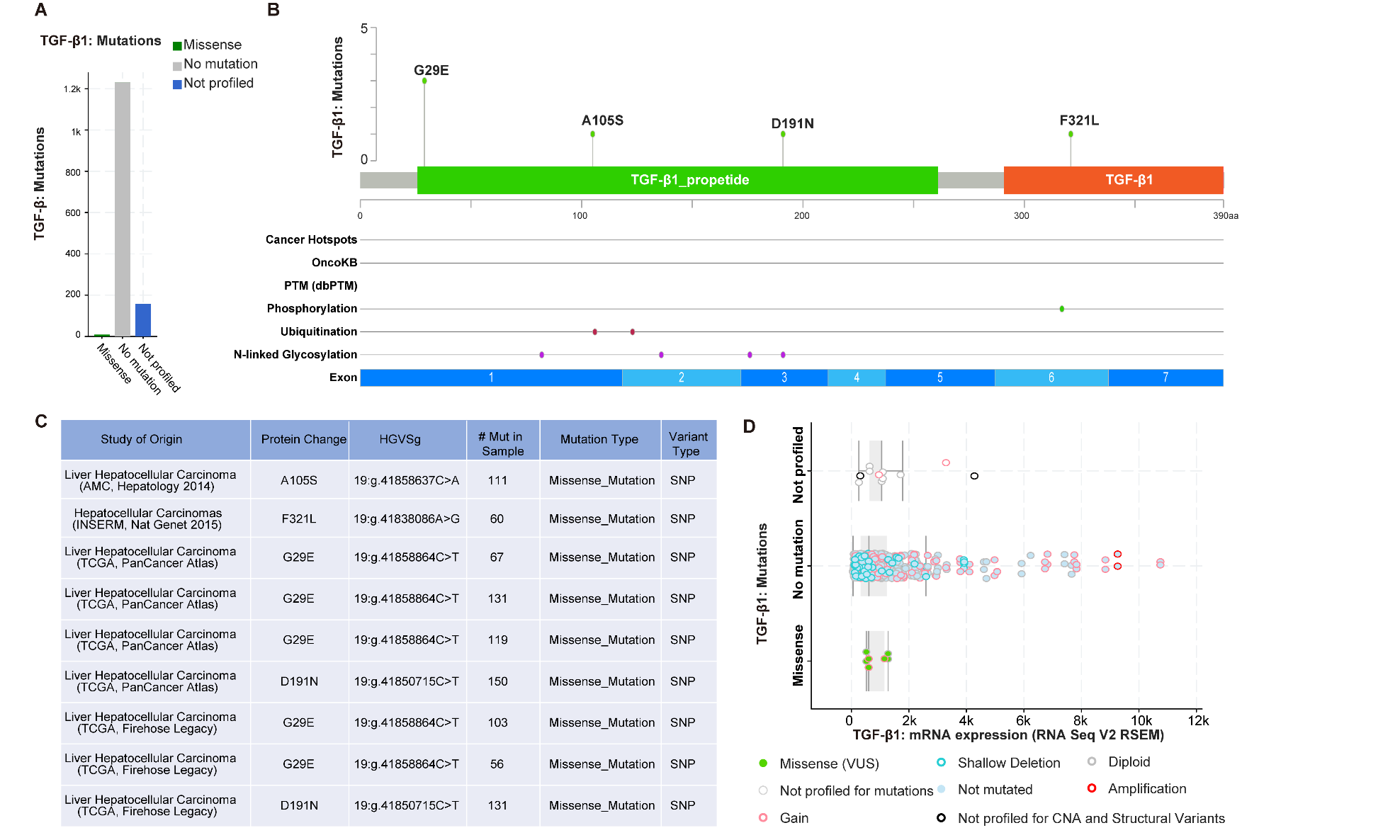


**Supplementary Figure 1.** ***TGF-β1* gene mutations in patients with HCC.** (A) *TGF-β1* mutation frequency in HCC; (B) *TGF-β1* mutation postition in HCC; (C) 11 *TGF-β1* Mutations: includes 4 duplicate mutations in patients with different HCC types; (D) *TGF-β1* mRNA vs mutation type in HCC patients.
